# Supplementary material for: Deletion of wfs1 Impairs Oligodendrocyte Precursor Cells Dorsal Distribution and Myelination Through the wfs1-hmgcs1 Axis in Zebrafish
Source: Biology (Basel). 2026 Aug 16;15(16):1402. doi: 10.3390/biology15161402 (PMC13510151; doi:10.3390/biology15161402)
Supplement: Supplementary file 1 [file biology-15-01402-s001.zip › File S1-Transcriptome sequencing data and differentially expressed genes (DEGs) in wfs1-- zebrafish at 5 dpf/1.GO/wfs1KOvsWT/GSEA/wfs1KO/RNA_CAP_BINDING/RNA_CAP_BINDING(GO_0000339).html]

Details for gene set RNA\_CAP\_BINDING(GO:0000339)[GSEA]


|  || Dataset | deg.sample |
| Phenotype | sample.cls |
| Upregulated in class | wfs1KO |
| GeneSet | RNA\_CAP\_BINDING(GO:0000339) |
| Enrichment Score (ES) | 0.643252 |
| Normalized Enrichment Score (NES) | 2.2198248 |
| Nominal p-value | 0.0 |
| FDR q-value | 0.012654717 |
| FWER p-Value | 0.023 |
Table: GSEA Results Summary

  

Fig 1: Enrichment plot: RNA\_CAP\_BINDING(GO:0000339)
  
Profile of the Running ES Score & Positions of GeneSet Members on the Rank Ordered List

  

| PROBE | DESCRIPTION   (from dataset) | GENE SYMBOL | GENE\_TITLE | RANK IN GENE LIST | RANK METRIC SCORE | RUNNING ES | CORE ENRICHMENT || 1 | 79380 | eif4ea |  |  | 35 | 4.922 | 0.3553 | Yes |
| 2 | 568761 | eif4g3b |  |  | 220 | 4.057 | 0.6433 | Yes |
| 3 | 336613 | cyfip1 |  |  | 10479 | 0.274 | 0.3413 | No |
| 4 | 192325 | ncbp2 |  |  | 11875 | 0.197 | 0.3118 | No |
| 5 | 493617 | eif4eb |  |  | 14120 | 0.086 | 0.2476 | No |
| 6 | 550549 | eif4e1c |  |  | 16678 | -0.022 | 0.1690 | No |
| 7 | 541523 | eif4e2 |  |  | 17083 | -0.036 | 0.1589 | No |
| 8 | 566131 | eif4g3a |  |  | 18648 | -0.106 | 0.1175 | No |
| 9 | 393112 | ncbp3 |  |  | 18755 | -0.111 | 0.1222 | No |
| 10 | 445157 | lsm1 |  |  | 22534 | -0.298 | 0.0252 | No |
| 11 | 336498 | eif3d |  |  | 22603 | -0.302 | 0.0450 | No |
| 12 | 447850 | eif4e3 |  |  | 23093 | -0.334 | 0.0538 | No |
| 13 | 393732 | eif4e2rs1 |  |  | 24066 | -0.397 | 0.0521 | No |
| 14 | 30738 | eif4e1b |  |  | 27314 | -0.650 | -0.0027 | No |
| 15 | 402850 | dcps |  |  | 27976 | -0.739 | 0.0300 | No |
| 16 | 100002872 | cyfip2 |  |  | 30090 | -1.280 | 0.0565 | No |
Table: GSEA details
[plain text format]

  

Fig 2: RNA\_CAP\_BINDING(GO:0000339)
  
Blue-Pink O' Gram in the Space of the Analyzed GeneSet

  

Fig 3: RNA\_CAP\_BINDING(GO:0000339): Random ES distribution
  
Gene set null distribution of ES for
 **RNA\_CAP\_BINDING(GO:0000339)**
